# Is There an Optimal Definition for a Positive Circumferential Resection Margin in Locally Advanced Esophageal Cancer?

**DOI:** 10.1245/s10434-021-10837-x

**Published:** 2021-10-07

**Authors:** Marius Kemper, Jakob R. Izbicki, Matthias Reeh

**Affiliations:** Department of General, Visceral and Thoracic Surgery, University Medical Centre Hamburg-Eppendorf, Hamburg, Germany

The aim of curative therapy is the complete resection of the esophageal tumor with a sufficient resection margin to healthy tissue. The more aggressive the tumor biology, the wider is the margin required to eliminate distant discontinuous infiltrating tumor cells. The anatomic proximity to organs that cannot be resected reasonably limits radical oncologic resection. These organs include the aorta, the trachea, and the spine. The maximum possible circumferential resection margin (CRM) thus depends on the stage of the disease at the time of resection. Despite radical surgical therapy, locoregional recurrence rates are reported to be as high as 52 %.^1,2^

In the majority of studies, the CRM has a prognostic impact on survival.^3^ However, studies also exist that could not establish a correlation between CRM and survival.^4^ Locoregional recurrence is the predominant failure pattern for patients with a positive CRM margin.^5^

Two different definitions of positive and negative CRM are used. The Royal College of Pathologists (RCP) defines a positive CRM as a tumor at or within 1 mm of the resection margin, whereas the College of American Pathologists (CAP) regards only the presence of tumor at the resection margins as CRM-positive.

The recently published study by Brac et al.^6^ included 283 patients retrospectively extracted from a prospectively maintained database. All the patients included in their study underwent transthoracic en bloc esophagectomy with curative intent for locally advanced (≥pT3, pNx) esophageal cancer, and 85.6 % of the patients underwent neoadjuvant treatment. More than two thirds (67.5 %) of the patients had lymphatic metastasis.

In the multivariable analysis, the following confounders were considered: pT, pN, preoperative treatment, sex, tumor location, American Society of Anesthesiologists (ASA) score, anastomotic site, histologic type, vascular invasion, and perineural invasion. The results showed that CRM, according to both definitions, was significantly associated with poor overall survival (OS) (CAP: hazard ratio [HR], 2.26, *p* < 0.001; RCP: HR, 1.42, *p* = 0.035). In addition, the authors showed that “a CRM=0 mm was predictive of a worse OS and DFS compared to a 0<CRM≤1 mm (*p* < 0.0001), whereas no significant difference was found between a CRM>1 mm and a 0<CRM≤ 1 mm, meaning that the CAP definition was more accurate to predict prognosis and recurrence.”

To determine the optimal CRM threshold value for predicting overall survival, the algorithm of the hazard ratio maximized with an incremental value of 100 µm was used. The authors propose 100 μm for patients with squamous cell carcinoma and 200 μm for patients with adenocarcinoma as a new cutoff value of CRM to predict overall survival optimally.

Despite the new findings of Brac et al.^6^ it remains the surgeon's aim to achieve maximal oncologic safety through a maximal radical circumferential resection margin. The study by Brac et al.^6^ does not answer the critical question: from what circumferential resection margin does the risk of locoregional recurrence no longer decrease? How far should a radical oncologic operation go?

Therefore, as a pragmatic approach, we propose that the circumferential distance in micrometers, without categorization, should be the standard in future pathologic reports, especially because in the near future the continuous evolution of multimodal therapy (e.g., the future use of immunotherapy^7^) very likely will change the optimal definition for a positive circumferential resection margin in locally advanced esophageal cancer. Especially, with regard to new upcoming multimodal treatment approaches, adenocarcinoma and squamous cell carcinoma must be examined separately in the future.

## References

[1] Law SYK, Fok M, Wong J (1996). Pattern of recurrence after oesophageal resection for cancer: clinical implications. Brit J Surg..

[2] Dresner SM, Griffin SM (2000). Pattern of recurrence following radical oesophagectomy with two-field lymphadenectomy. Brit J Surg..

[3] Karstens K-F, Izbicki JR, Reeh M (2017). Does the margin matter in esophageal cancer. Digest Surg..

[4] Ghadban T, Reeh M, Koenig AM (2017). Prognostic significant or not? The positive circumferential resection margin in esophageal cancer: impact on local recurrence and overall survival in patients without neoadjuvant treatment. Ann Surg..

[5] Park HJ, Kim HJ, Chie EK (2013). The influence of circumferential resection margin status on locoregional recurrence in esophageal squamous cell carcinoma. J Surg Oncol..

[6] Brac B, Dufour C, Behal H (2021). Is there an optimal definition for a positive circumferential resection margin in locally advanced oesophageal cancer?. Ann Surg Oncol..

[7] Kelly RJ, Ajani JA, Kuzdzal J (2021). Adjuvant nivolumab in resected esophageal or gastroesophageal junction cancer. N Engl J Med..

